# Dazzled by shine: gloss as an antipredator strategy in fast moving prey

**DOI:** 10.1093/beheco/arad046

**Published:** 2023-06-08

**Authors:** Patricia Henríquez-Piskulich, Devi Stuart-Fox, Mark Elgar, Ivan Marusic, Amanda M Franklin

**Affiliations:** School of BioSciences, The University of Melbourne, Parkville, Victoria 3010, Australia; School of BioSciences, The University of Melbourne, Parkville, Victoria 3010, Australia; School of BioSciences, The University of Melbourne, Parkville, Victoria 3010, Australia; Department of Mechanical Engineering, The University of Melbourne, Parkville, Victoria 3010, Australia; School of BioSciences, The University of Melbourne, Parkville, Victoria 3010, Australia

**Keywords:** dynamic dazzle, flash coloration, gloss, motion dazzle, predation, protective coloration, specular reflectance

## Abstract

Previous studies on stationary prey have found mixed results for the role of a glossy appearance in predator avoidance—some have found that glossiness can act as warning coloration or improve camouflage, whereas others detected no survival benefit. An alternative untested hypothesis is that glossiness could provide protection in the form of dynamic dazzle. Fast moving animals that are glossy produce flashes of light that increase in frequency at higher speeds, which could make it harder for predators to track and accurately locate prey. We tested this hypothesis by presenting praying mantids with glossy or matte targets moving at slow and fast speed. Mantids were less likely to strike glossy targets, independently of speed. Additionally, mantids were less likely to track glossy targets and more likely to hit the target with one out of the two legs that struck rather than both raptorial legs, but only when targets were moving fast. These results support the hypothesis that a glossy appearance may have a function as an antipredator strategy by reducing the ability of predators to track and accurately target fast moving prey.

## Teaser

Animals with glossy surfaces are ubiquitous in nature, but why these striking appearances have evolved is unclear. We tested whether gloss provides protection from predators when prey are moving. Praying mantids were less likely to strike at moving targets that were glossy rather than matte, and were less likely to track and accurately strike glossy targets when they were moving fast. This study provides the first empirical evidence that gloss could offer protection against predation for fast moving animals.

## INTRODUCTION

Organisms with glossy surfaces can be found throughout the tree of life, from beetles, bees and spiders, to birds, reptiles and even flowers (Seago et al. 2009; Whitney et al. 2012; van der Kooi et al. 2017; Whyte and Anderson 2017; Eliason and Clarke 2020; Crowe-Riddell et al. 2021). Glossy describes a visual effect produced by smooth surfaces that reflect a high proportion of light at the specular or mirror angle, equal and opposite to the angle of illumination (Toomey et al. 2010; Maia et al. 2011; Papiorek et al. 2014; Igic et al. 2015; Franklin and Ospina-Rozo 2021). This visual effect is subjective and influenced by viewer perception in addition to surface properties and viewer or illumination geometry (Chadwick and Kentridge 2015). It can be distinguished from the surface optical property of “gloss” measured as the proportion of light reflected at the specular angle (Franklin and Ospina-Rozo 2021). Gloss differs from iridescence because it relates to an angle-dependent change in light intensity (specularity); whereas iridescence refers to an angle-dependent change in hue (Stuart-Fox et al. 2020). The visual appearance of gloss is dynamic because it changes with the angle of illumination and/or observation, which occurs when there is movement. Given its ubiquity, gloss could have both visual and non-visual functions, such as communication and camouflage or thermoregulation and water repellence (Chapman 2013; Wang et al. 2021; Ospina-Rozo et al. 2022). Although visual functions of gloss were proposed several decades ago (Hinton 1973; Neville 1977), only recently have these predictions begun to be tested. Gloss can amplify warning signals (Waldron et al. 2017), and potentially enhance survival by having an aversion effect on predators (Kjernsmo et al. 2022). It also works as a form of camouflage by background matching (reflecting surroundings) in some animals (e.g., marine fish, Denton 1971; Johnsen et al. 2014), whereas in others it appears not to (e.g., beetles, Franklin et al. 2022). These previous studies have only investigated gloss as an antipredator strategy in stationary prey. As the appearance of gloss is dynamic, incorporating movement could provide important insight into its function.

Animal movement is often nonlinear, their bodies change position to accommodate to the surface they are walking on or other environmental variables (e.g., wind, obstacles). As they move, the angle between different body parts, the sun and the observer changes, potentially producing flashes as the animal moves. Thus, it would be expected that fast moving glossy animals would produce flashes of light that increase in frequency at higher speeds, creating an ever-changing pattern. This could resemble what has been previously described as motion dazzle—high-contrast animal color patterns that can inhibit capture by reducing the ability of predators to correctly assess speed and trajectory (Thayer 1909; Stevens and Merilaita 2009). These benefits of motion dazzle are affected by prey speed, with reduced capture success and distortion of speed perception only observed at intermediate or fast speeds (Stevens et al. 2008; Scott-Samuel et al. 2011; Kodandaramaiah et al. 2020). In addition, Hall et al. (2016) showed that dynamic patterns (i.e., a moving black and white sinusoidal grating) have a greater effect on perceived speed than static patterns (i.e., stationary black and white sinusoidal grating), reducing or increasing the perceived speed of a target depending on the direction of motion of the pattern. Studies of dynamic patterns have also investigated iridescence (i.e., changes in hue with change in illumination or viewing angles) and flash coloration (i.e., where appearance changes between two colors as the animal moves, such as beating wings of butterflies and birds if the upper and lower surfaces differ in color). These types of display reduce capture success and attack accuracy of predators (Pike 2015; Murali 2018; Murali and Kodandaramaiah 2020), particularly at a higher change in the frequency of the patterns (Murali 2018). Glossiness could also function as a dynamic pattern to help moving prey avoid predators by producing flashes of light, or by producing high contrast patterns similar to motion dazzle. It has also been proposed that glossiness may mislead predators about prey shape, position, and/or speed because the perceived depth of the specular reflection differs from the depth of the reflecting surface for stereoscopic vision (Nityananda and Read 2017; Adams et al. 2019). Whether glossiness improves predator avoidance for moving prey has never been tested with realistic targets or real predators.

Praying mantids are visual predators that attack moving prey. The movement perception of these insects is well studied, including their use of binocular stereopsis for depth perception and their use of motion cues to target prey (Nityananda et al. 2015, 2016, 2018, 2019). Praying mantids tend to be generalist predators and experiments with artificial targets demonstrate that a diverse range of target characteristics are recognized as prey (Rilling et al. 1959; Prete et al. 2011, 2012, 2013). Many of their prey items (e.g., bees, flies, dragonflies) are glossy and fast moving. Current evidence suggests that preferred prey size depends on praying mantid body size (Iwasaki 1991), with higher striking rates occurring at higher speeds (Prete et al. 2011). Praying mantids are also capable of modifying their strike behavior in relation to prey speed, approaching more quickly and without pauses when prey are faster (Rossoni and Niven 2020). They likely have monochromatic vision (Sontag 1971; Kral and Prete 2004), meaning they can only perceive intensity differences (i.e., brightness), not color variation, which is useful to isolate the effect of achromatic from chromatic prey characteristics. Therefore, mantids are ideal to investigate whether glossiness impacts attack behaviors.

In this study, we used the giant rainforest mantis (*Hierodula majuscula*Tindale 1923) to test how glossiness and speed interact to affect the prey capture effectiveness of predators. We placed mantids in an arena with a matte background and a directional light source to maximize the glossy appearance of targets. Mantids were presented with matte or glossy targets of the same hue at two different speeds. We documented tracking, number of strikes, success, accuracy, and latency parameters. We predicted that at higher speed, glossy targets would be tracked less often, struck less and less successfully, and with lower strike accuracy and longer latency than matte targets.

## METHODS

### Predators

Experiments were carried out with 26 female mantids of the species *H. majuscula* purchased from Minibeast Wildlife (Kuranda, QLD, Australia), where they were reared in captivity. In the laboratory, mantids were kept individually in cages made from mesh (30 × 30 × 30 cm) at 27°C. They were sprayed with water once a day and fed two times a week with wood cockroaches (*Nauphoeta cinerea*Olivier 1789) purchased from Minibeasts Enterprises (Bannockburn, VIC, Australia). Experiments were conducted between 27 March 2022 and 17 April 2022.

### Targets

**Figure 1:**
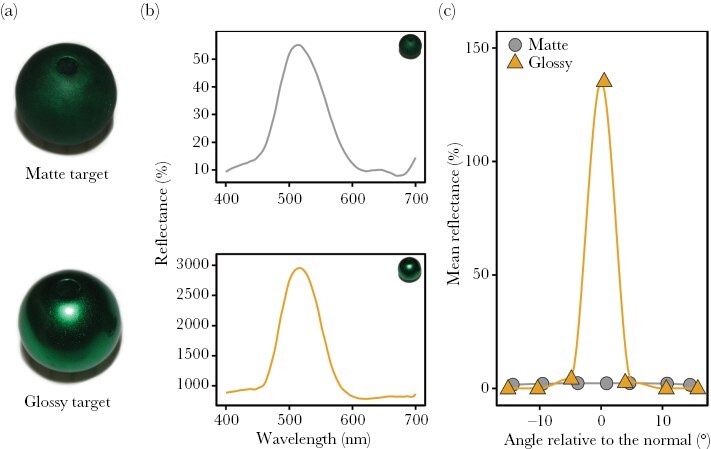
Optical properties of targets used for the experiment. (a) Targets. (b) Spectral reflectance of targets. Data are plotted as a percentage relative to the reflectance of a diffuse standard (i.e., calibrated against a Lambertian 99% reflectance spectralon standard). The glossy target has a reflectance value above 100% because it reflects more light at the specular angle than the diffuse standard. The specular or mirror angle is where the angle of reflection equals the angle of incident light in relation to the surface normal. (c) Angular change in reflectance of targets. Mean reflectance at the specular or mirror angle and shifting away from the specular angle. The glossy target had high mean reflectance at the specular angle and low mean reflectance at all other angles, indicating high specularity, whereas the matte target showed minimal angle dependency in reflectance, indicating low specularity.

To approximate glossy and matte prey the targets were made from 14 mm smooth acrylic beads, with matte targets finely sanded to remove the smooth surface and therefore substantially reduce glossiness. All targets were airbrushed with chrome silver 4107 (Alclad II Lacquer, USA), followed by a layer of clear green acrylic paint PL20 (SMS, Australia). We chose green for the targets (Figure 1b) to ensure that mantids could perceive the achromatic changes, as research suggests that mantids have one green-sensitive photoreceptor with a peak sensitivity (λ_max_) between 510 and 520 nm (Sontag 1971; Rossel 1979). To enhance gloss differences, glossy targets and matte targets were airbrushed with a layer of high gloss paint PL58 or flat clear coat acrylic paint PL10 (SMS, Australia), respectively (Figure 1a). Our approach to create glossy and matte targets was based on Franklin et al. (2022), who showed that the difference in gloss produced closely matches the difference between glossy and matte green beetles in their natural habitat, where *H. majuscula* is also found (rainforest of north-eastern Australia). The treatments are therefore within the range of glossiness observed among insect species.

We quantified the difference in gloss between treatments (glossy and matte) following the methods described by Gruson et al. (2019) and Ospina-Rozo et al. (2022), as well as by using a glossmeter. For both measurements, a flat sample provides more accurate measurements of gloss. Therefore, instead of measuring the spherical targets, flat acrylic squares (2 × 2 cm) were prepared following the same methodology described above. First, we measured reflectance using an Ocean Optics USB 2000+ spectrometer and PX-2 pulsed xenon light source, calibrated with a diffuse spectralon standard (Ocean Optics, USA). These were coupled to a goniometer enabling measurement at a precise set of geometries. To measure angular changes in mean reflectance (i.e., specularity), independently of angular changes in wavelength (i.e., iridescence), we fixed the angular span between the light source and collector at 20° and the bisector between the light source and collector was shifted to different angles from the normal: −30°, −20°, −10°, 0°, 10°, 20°, and 30°. The angle 0° represents the specular or mirror angle, which is where the angle of reflection equals the angle of incident light in relation to the surface normal, an imaginary line perpendicular to the surface of an object. Other angles are offset from the specular angle. High gloss surfaces will reflect more light at the mirror angle compared to other angles, whereas low gloss surfaces will have a more even spread of light reflected across all angles. For each measurement geometry, we took three measurements and used the average. We then fitted a Gaussian curve to the plotted mean reflectance values for the set of measurement geometries of a given treatment sample (glossy or matte; Figure 1c) (Gruson et al. 2019; Ospina-Rozo, Roberts, et al. 2022). Specularity was calculated as the inverse of the width of the Gaussian curve, where higher values indicate a smaller angular spread of light reflected away from the specular or mirror angle and therefore higher gloss (Franklin et al. 2022; Ospina-Rozo et al. 2022). Glossy targets had high specularity (specularity = 1.73), whereas matte targets had very low specularity (specularity = 0.06).

Second, we quantified the differences in specular reflectance between the targets by using a ZGM1120 glossmeter (Zehntner Testing Instruments, Switzerland) and the software GlossTools v1.0.0050. Glossmeters measure gloss in gloss units (GU), which is the proportion of light reflected relative to a black glass standard (GU = 100). Glossmeters measure the proportion of light reflected at specific angles and are commonly used to measure gloss in visual ecology research (Papiorek et al. 2014; Kjernsmo et al. 2020, 2022). Following the manufacturer recommendations, we measured the glossy treatment at 20° and the matte treatment at 60° to optimize measurement accuracy. Three readings were taken and averaged for each treatment. The readings showed that glossy and matte treatments had 129.83 and 0.49 GU, respectively.

### Experimental design

**Figure 2:**
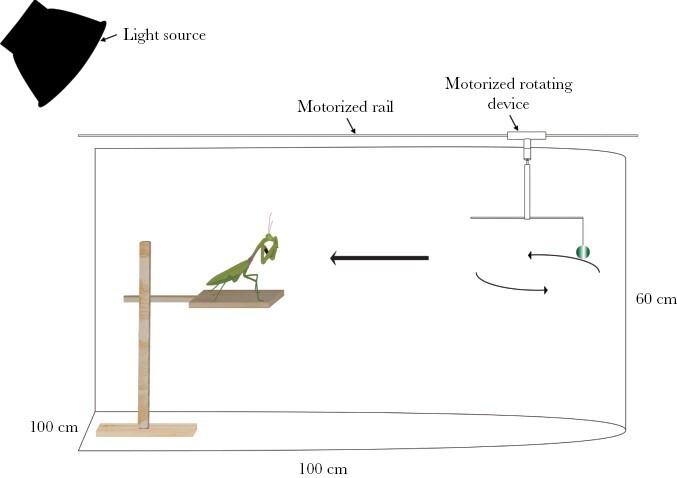
Diagram of the experimental set-up. Mantids were placed on a platform (8 × 8 cm) of 32 cm height at the edge of the semicircular arena facing the targets, which were mounted in a controlled rotating device located at the other end of the arena attached to a motorized rail. Targets had a diameter of 14 mm.

Experiments were carried out in a curve-walled arena, with base dimensions 100 cm × 100 cm and walls 60 m high (Figure 2), and with a 21.28% mean reflectance matte gray background. To assess the mantids’ ability to capture moving glossy and matte prey, mantids were placed at the edge of the arena on a platform which consisted of a stand holding a wooden perch of 8 cm × 8 cm at a height of 32 cm from the arena floor. On the opposite edge of the arena and facing the mantids, targets were hung from a motorized rotating device (4 cm distance from the axis of rotation and 4 cm height above the perch) attached to a motorized rail on top of the arena, to control speed. The rotating device was controlled using a Powertech 12VDC 8A motor speed controller MP3209. The rail was controlled through a script run in Arduino IDE 1.8.19 (Arduino, Italy). The arena was illuminated from the side closest to the platform, using an Aputure Amaran 100x Bi-Color LED light, which replicates the color temperature of daylight (6,500K, CRI ≥ 95). Direct light at a 30% intensity was located to the left side of the rail at approximately 80 cm and at a 45° angle to produce constant illumination over the target and enhance the specular reflectance viewable by the mantid.

Speed was manipulated by adjusting both the rail speed and target rotational speed. Attaching the rotating device on the motorized rail allowed us to control the speed at which the targets traveled toward the platform from the opposite edge of the arena in a linear motion, while the rotating device controlled the rotational speed of the target. We considered two speeds: (1) 36 rpm (velocity 15.08 cm/s; angular velocity 3.77 rad/s) and rail speed of 11.4 cm/s, which we refer to as “slow”; and (2) 52 rpm (velocity 21.8 cm/s; angular velocity 5.45 rad/s) and rail speed of 17.3 cm/s, which we refer to as “fast.” These speeds were chosen to be within the likely range of movement speeds for prey of *H. majuscula*. Mantids are generalists and opportunistic predators that feed primarily on arthropods (Reitze and Nentwig 1991; Kral and Prete 2004), but can also feed on small invertebrates (Kolnegari et al. 2022). They are capable of attacking a range of walking or flying prey that move at different speeds, and capable of adjusting their strike accordingly (Prete et al. 1993; Rossoni and Niven 2020). For example, flies such as *Drosophila* spp. (which can be a food source for mantids in captivity) fly between 30 cm/s and 200 cm/s (Dean 2003). Given that our protocol included both circular and forward movement, speeds chosen were based on pilot trials to make the task sufficiently difficult but not so difficult that mantids would miss all strikes. This placed our fast treatment within the low end of the range of *Drosophila* spp. flight speeds. Every individual of *H. majuscula* was presented with four different treatments: (1) Glossy and slow target; (2) matte and slow target; (3) glossy and fast target; and (4) matte and fast target. For all treatments, the rotating device travelled 70 cm and stopped once the target was above the platform, continuing to rotate for 5 s around the mantis before the rail returned to the starting point, ending the trial.

Each mantid completed all four experimental treatments and was assigned to one of four pseudorandomised groups. To control for learning, the order of treatments was alternated to ensure roughly equal numbers of mantids were exposed to each treatment for the first, second, third, and fourth trials (i.e., balanced among treatment groups; Supplementary Table S.1). Mantids were given a week between treatments to allow them to forget previous experiences. To avoid habituation, we also fed the mantids a cockroach (*N. cinerea*) attached to the rotating device in the arena between trials (Maldonado 1972; Maldonado et al. 1979). Cockroaches were presented in a way that replicated the treatment movement used in the experiment, with a circular and forward movement. Mantids were not fed for three days prior to each treatment presentation, to maintain motivation.

Trials were recorded using a Photron FASTCAM NOVA S16 High Speed Camera placed on the right side of the arena at 1,000 fps with a shutter speed of 1/7,000. We calibrated pixel size with the software PFV4 (×64) and using a measuring tape on the side of the rail that was facing the camera. From the videos, we extracted the following response variables: (1) Tracking (binary)—defined as turning of the head and/or prothorax toward the target after motion commenced and head movements that followed the movement of the target; (2) Total number of strikes (count)—for the mantids that tracked the target, the number of times they attempted to strike the target, whether successful or unsuccessful; (3) Proportion of success (proportion)—defined as the proportion of strikes out of the total number of strikes that ended with the target being displaced by having contact with the spines of the femur and/or tibia of one or both raptorial legs, only measured for mantids that attacked at least once; (4) Strike accuracy (binary)—mantids always strike prey with their two raptorial legs, therefore, for individuals that successfully hit a target, accuracy was measured as whether they hit the target with one or two raptorial legs because hitting with one leg is more likely to result in an unsuccessful strike with the prey escaping; (5) Latency to strike (continuous)—to assess whether the mantids waited for the rotating device to stop on top of the platform before attacking, when the rotating device stopped it was considered zero seconds, and negative values represented attacks that occurred before the rotating device stopped, time of strike was recorded as when the mantis initiated the strike movement. Data extraction from videos was undertaken by one person (P.H.-P.) and was not blinded because treatments were clearly visible. Video examples of successful and unsuccessful strikes are provided in the Supplementary Material.

### Statistical analyses

Data analyses were conducted in R 4.2.0 (R Core Team 2022), with the packages lme4 (Bates et al. 2015), bbmle (Bolker and R Core Team 2017), car (Fox and Weisberg 2019), and tidyverse (Wickham et al. 2019). Data were analyzed with linear mixed models for continuous data and generalized linear mixed models (GLMMs) with binomial or Poisson distribution for binary, proportion, and count data. We ran models for the five response variables previously described. All models included the interaction between treatment (glossy or matte) and speed (slow or fast), and trial order (continuous) as fixed effects and mantid ID as a random effect. To determine whether trial order explained significant variation in the data, reduced models were compared to full models using AIC (Zuur et al. 2009). Fit of all models was assessed using diagnostic plots and models did not violate assumptions. The model assessing total number of hits was tested for overdispersion given that the response variable consisted of count data. This was done by calculating the sum of squared Pearson residuals and then comparing this sum to the residual degrees of freedom (Zuur et al. 2009); no overdispersion was detected (*P*-value = 0.979). *P*-values of the explanatory variables of selected models were obtained through a Wald Chi-Squared test. We consider *P*-values to be a continuous measure of statistical evidence rather than using an arguably arbitrary *P* < 0.05 significance cut off (Kirk 1996; Greenland et al. 2016; Muff et al. 2022). For GLMMs, maximum likelihood estimates (MLEs), effect size, and confidence interval (CI) estimates were used to assess differences between speed, treatment and/or their interaction (Nakagawa and Cuthill 2007; Halsey et al. 2015). This provides an indication of the magnitude of differences among treatment groups and the uncertainty in our estimates.

## RESULTS

### Tracking

**Figure 3:**
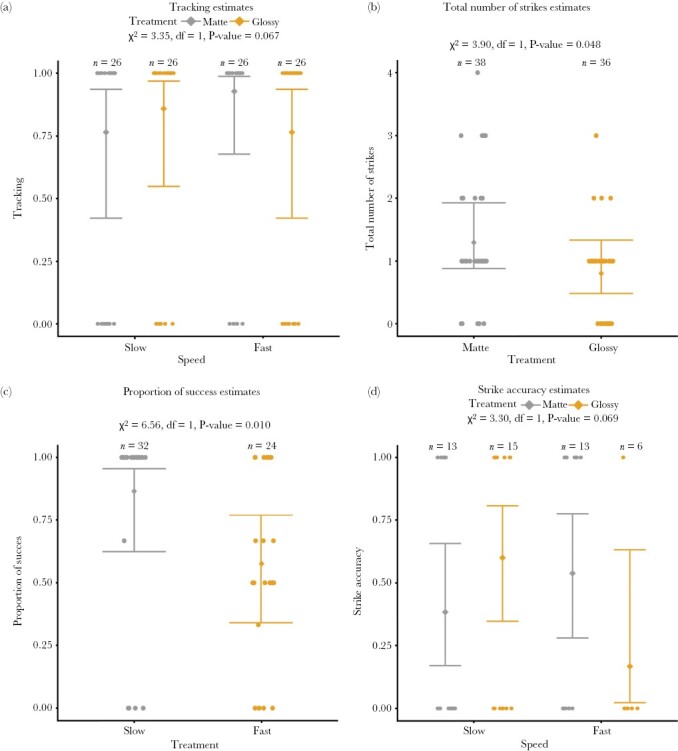
Mean and 95% CI estimated from the regression models. Dots represent the observed results of each trial. See Supplementary Figure S.1 for unpooled plots. (a) Weak evidence of less tracking for targets that were glossy and fast (interaction term: χ^2^ = 3.35, df = 1, *P*-value = 0.067). (b) Moderate evidence that total number of strikes was lowest for glossy targets, regardless of speed (χ^2^ = 3.90, df = 1, *P*-value = 0.048). (c) Strong evidence that proportion of successful strikes is lowest at higher speed (χ^2^ = 6.56, df = 1, *P*-value = 0.010). (d) Weak evidence of lower strike accuracy for targets that were glossy and fast (interaction term: χ^2^ = 3.30, df = 1, *P*-value = 0.069).

The experiment included trials for 26 individuals (*n* = 104 trials), for which we found weak evidence that the effect of glossy and matte targets on target tracking differed depending on target speed (interaction term: χ^2^ = 3.35, df = 1, *P*-value = 0.067; Figure 3a). The interaction is driven by opposite effects of glossiness at slow (mean, 95% CI; glossy: 86%, 55–97%; matte: 77%, 42–94%) versus fast speeds (mean, 95% CI; glossy: 77%, 42–94%; matte: 93%, 68–99%; Table 1). Mantids were 9% more likely to track glossy slow targets compared to matte slow targets; whereas they were approximately 16% less likely to track glossy fast targets compared to matte fast targets, although in both cases, 95% CIs overlapped substantially. This suggests that glossy targets may be more difficult to track at fast speeds, although the evidence is weak.

**Table 1:** Effects of treatment and speed on mantis behavior

Response variable	MLE (95% CI)				Probability distribution	Explanatory variable	*χ2*	df	*P* value
	Matte and slow	Glossy and slow	Matte and fast	Glossy and fast					
Tracking	0.77 (0.42–0.94) *n* = 26	0.86 (0.55–0.97) *n* = 26	0.93 (0.68–0.99) *n* = 26	0.77 (0.42–0.94) *n* = 26	Binomial	Treatment	0.09	1	0.769
						Speed	0.49	1	0.482
						**Treatment:Speed**	**3.35**	**1**	**0.067**
						**Trial order**	**6.62**	**1**	**0.010**
Total number of strikes	1.12 (0.71–1.75) *n* = 17	0.95 (0.60–1.50) *n* = 19	1.48 (1.04–2.10) *n* = 21	0.65 (0.36–1.17) *n* = 17	Poisson	**Treatment**	**3.90**	**1**	**0.048**
						Speed	0.03	1	0.876
						Treatment:Speed	1.88	1	0.170
Proportion of success	0.79 (0.55–0.92) *n* = 16	0.94 (0.69–0.99) *n* = 16	0.52 (0.35–0.68) *n* = 16	0.64 (0.34–0.86) *n* = 8	Binomial	Treatment	1.59	1	0.208
						**Speed**	**6.56**	**1**	**0.010**
						Treatment:Speed	0.54	1	0.461
Strike accuracy	0.39 (0.17–0.66) *n* = 13	0.60 (0.35–0.81) *n* = 15	0.54 (0.28–0.78) *n* = 13	0.17 (0.02–0.63) *n* = 6	Binomial	Treatment	0.04	1	0.852
						Speed	0.06	1	0.800
						**Treatment:Speed**	**3.30**	**1**	**0.069**
Strike latency	1.70 (0.91–2.49) *n* = 16	1.98 (1.19–2.77) *n* = 16	1.80 (1.01–2.60) *n* = 16	2.75 (1.66–3.84) *n* = 8	Log-normal	Treatment	1.87	1	0.172
						Speed	0.79	1	0.374
						Treatment:Speed	0.68	1	0.411

Relevant results of Wald Chi-Squared test for the model are highlighted in bold.

Trial order was only included if it explained significant variation in the data when reduced models were compared to full models using AIC.

Trial order affected tracking behavior (χ^2^ = 6.62, df = 1, *P*-value = 0.010), indicating that mantids engaged more with the experiment with each trial they underwent. Tracking was the only behavior for which treatment order explained some of the variation; therefore, treatment order was not included in the final model for other behaviors (Table 1).

### Number of strikes

Glossiness influenced the total number of strikes, regardless of speed (χ^2^ = 3.90, df = 1, *P*-value = 0.048; Figure 3b). Total number of strikes was 0.5 lower for glossy targets in comparison to matte targets (glossy: 0.8, 95% CI: 0.5–1.3, on average; matte: 1.3, 95% CI: 0.9–1.9, on average), suggesting that mantids are less likely to strike glossy targets. We found no evidence that the interaction between speed and treatment affected the total number of strikes (interaction term: χ^2^ = 1.88, df = 1, *P*-value = 0.170).

### Proportion of successful strikes

The proportion of successful strikes was influenced by speed (χ^2^ = 6.56, df = 1, *P*-value = 0.010) (Figure 3c). The proportion of successful strikes was 29% higher for slow targets compared with fast targets (slow: 87%, 95% CI: 62–96%, on average; fast: 58%, 95% CI: 34–77%, on average), indicating that mantids were less successful when striking fast targets. We found no evidence that the interaction between speed and treatment had an effect on the proportion of successful strikes (interaction term: χ^2^ = 0.54, df = 1, *P*-value = 0.461).

### Strike accuracy

We found some indication that the effect of glossiness on strike accuracy differed depending on target speed (interaction term: χ^2^ = 3.30, df = 1, *P*-value = 0.069; Figure 3d). Similar to tracking, the treatment effect was opposite at slow (glossy: 60%, 95% CI: 35–81%; matte: 39%, 95% CI: 17–66%) and fast speeds (glossy: 17%, 95% CI: 2–63%; matte: 54%, 95% CI: 28–78%). Mantids were 21% more likely to strike glossy slow targets with two raptorial legs compared to matte slow targets, but 37% less likely to strike glossy fast targets with two raptorial legs compared to matte fast targets, although confidence intervals overlap substantially. This suggests that mantids might be more likely to strike glossy and fast targets with one raptorial leg instead of two.

### Strike latency

Finally, we found no evidence that treatment, speed, or the interaction between them influenced strike latency (*P*-values >= 0.172 in all cases; Table 1).

## DISCUSSION

We tested the hypothesis that high gloss may reduce predation risk for fast moving prey. Our results suggest that mantids may be less likely to track and attack fast glossy targets, irrespective of their speed. When mantids attempted to strike the target, there was no difference in latency of attack between glossy and matte or fast and slow targets, but there was a lower proportion of successful strikes for fast targets, independently of being glossy or matte. When mantids successfully struck the target, their accuracy (estimated as whether they struck with one or both raptorial legs) tended to be lower for fast moving glossy prey than fast moving matte prey. These results suggest that a glossy appearance may provide an advantage for prey by reducing the probability of attack by predators, and possibly reducing capture success. This experiment is the first to provide evidence of possible advantages of glossiness in fast moving prey, by reducing the probability of tracking and attacking, and therefore increasing the chances of surviving a predator encounter.

There are several reasons why mantids may be less likely to track fast moving glossy prey and less likely to attack glossy prey in general. First, glossiness may reduce the detectability of prey. Mantids use brightness differences for depth perception and to detect motion at a broad range of speeds (Nityananda et al. 2015, 2018). Thus, glossy prey may be easier to detect as high gloss generates brightness contrast and brightness changes in moving prey. However, a high contrast pattern reduces the neurological response of locusts to looming stimuli (Santer 2013). It is possible that if glossy prey produce high contrast patterns, these may similarly impact the neurological response of mantids. Further research into the visual processing of glossy stimuli may uncover interesting results. Second, glossy targets may be less likely recognized as prey once detected. However, this also seems unlikely as mantids attacked the targets in the majority of experiments and the speeds defined as fast for this experiments for both the rotating device (52 rpm or 21.8 cm/s) and the rail (17.3 cm/s) are in the slower range of described insect speeds (Dean 2003). A third explanation for less tracking of glossy fast targets is that the mantids avoided breaking camouflage to try to capture difficult prey. Glossy targets produce intense flashes of light with movement, and could create a pattern that makes prey harder to accurately localize depending on their speed (Umeton et al. 2019). Mantids are typically sit-and-wait predators and employ crypsis to hunt for their prey (but see Bertsch et al. 2019). It is possible that fast moving glossy targets are more difficult to capture than fast moving matte targets, and breaking camouflage might not be worthwhile if the prey is too hard to catch. This is also reflected in the results for the total number of strikes, where glossy targets were attacked less often, or not at all, than matte targets.

When mantids attempted to strike the target, there was no difference in their latency to strike but, as expected, they were less likely to successfully strike fast targets compared to slow targets. The proportion of successful strikes did not differ for glossy and matte targets, but we found weak evidence that glossiness could reduce predator strike accuracy for fast moving prey, as mantids more frequently struck glossy fast prey with one raptorial leg out of the two that struck. Here we considered successful strikes as any contact between the target and the spines of the femur and/or tibia of one or both raptorial legs; however, this may not correspond to successful capture of live (likely struggling) prey, particularly if the prey is captured in only one raptorial leg. Thus, it is possible that the higher probability of striking fast and glossy prey with only one raptorial leg could decrease the probability of successfully capturing and subduing prey, increasing the probability of prey survival.

Together, our results suggest that a glossy appearance may act as dynamic dazzle and improve prey survival, particularly at fast speeds. Glossiness may act as dynamic dazzle (i.e., moving patterns or flashes on moving prey), reducing capture success and attack accuracy of predators (Pike 2015; Murali 2018), and doubling the mismatch between perceived speed and actual speed compared to static patterns (Hall et al. 2016). Mantids do not use binocular disparity to compare brightness between the two eyes for stereopsis like vertebrates, but rather look for areas where brightness is changing independently, outperforming vertebrate depth perception (Nityananda et al. 2018). Despite their more efficient judgement of depth, mantids seem to be less responsive to high contrast patterns (Umeton et al. 2019). Glossiness creates high contrast dynamic patterns that could have the same effect. Here, we used physical targets and animal predators to demonstrate that glossy surfaces may also act as dynamic dazzle, likely through flashes produced during motion (disco-ball effect). At faster speeds, these flashes will occur at higher frequency. Flash coloration is more effective at higher change frequency (Murali 2018) so this change in flash frequency may contribute to poorer mantid performance at faster speeds. It is also possible that the spatial pattern produced by highly reflective, glossy targets impacts speed perception and capture success at intermediate or fast speeds, similar to prey with static patterns (Stevens et al. 2008; Scott-Samuel et al. 2011; Kodandaramaiah et al. 2020). Disentangling temporal changes from spatial variation will reveal whether both components contribute to prey survival and identify specific prey characteristics that impact capture success.

An important consideration is that color patterns often need to provide protection from predators when prey are both static and moving (Tan and Elgar 2021). When prey are static, the high contrast patterns that create motion dazzle when prey are moving (e.g., black and white stripes; Stevens et al. 2011; Hughes et al. 2014; Hogan et al. 2016) could provide disruptive camouflage when prey are static (Schaefer and Stobbe 2006; Stevens et al. 2006; Webster et al. 2013). Similarly, it is possible that glossy surfaces provide protection due to dynamic dazzle when prey are moving but enhance protection through other mechanisms (e.g., aposematism or background matching (Waldron et al. 2017; Kjernsmo et al. 2020, 2022) when prey are static. Alternatively, glossy surfaces may only provide an advantage when prey are moving (or only to fast moving prey) (Stevens et al. 2011; Franklin et al. 2022). These mirror-like surfaces would produce greater temporal and spatial visual changes whilst in motion than less glossy surfaces, which could increase the benefits of dynamic dazzle. Therefore, there may be an association between prey “glossiness” and movement behavior (e.g., proportion of time spent moving, average speed), which could be tested using a comparative approach.

The perception of gloss is multidimensional and other cues such as background and illumination can affect how glossy objects are perceived (Chadwick and Kentridge 2015). This experiment only considered the interaction between glossy and matte appearances with speed, but other factors could also play a role in the efficacy of gloss as an antipredator strategy (Franklin 2022). For example, the perception of flashes of light produced by glossy cuticles could also be affected by the visual background. Highly glossy or densely vegetated backgrounds, such as in tropical forests, could create more visual contrast and noise, and interact with gloss to reduce the predator’s ability to discriminate, accurately locate, and target prey (Xiao and Cuthill 2016; Kjernsmo et al. 2020). Additionally, dynamic lighting environments could simultaneously enhance the dynamic dazzle of glossy fast-moving prey, and complex backgrounds by adding changes in motion, luminance, and edge noise, making it harder to capture moving prey (Matchette et al. 2018, 2019; Cuthill et al. 2019). This dynamic lighting could add an extra layer of complexity to dynamic dazzle patterns of glossy fast prey, making them harder to locate compared with constant lighting conditions. Alternatively, predators with polarization vision could minimize the negative impact of dynamic lighting and be able to locate targets (Venables et al. 2022). On the other hand, in simple backgrounds such as open habitats, prey glossiness could have the opposite effect and predators might be able to detect and locate glossy moving prey more easily. Our understanding of how predator visual systems perceive complex visual scenes and how they contribute to antipredator strategies is far from complete (Franklin 2022), and there is significant scope to further explore the role of glossiness in protection from predators.

Our study provides evidence that a glossy appearance may improve prey survival, particularly for fast moving prey. By creating precisely controlled targets, we were able to isolate the effect of glossiness on predator attack behaviors and provide support for predictions that a glossy appearance plays a role in predator avoidance for moving prey (Thayer 1909; Franklin et al. 2022). However, this does not necessarily indicate that gloss evolved as an antipredator strategy. Gloss likely has multiple functions, including both visual (e.g., communication or camouflage) or non-visual functions. For example, highly glossy surfaces may play a role in thermoregulation by reducing heating rates, if they reflect a high proportion of incident light (Wang et al. 2021; Ospina-Rozo et al. 2022), or by reflecting light to specific organs that require precise temperatures to function (van der Kooi et al. 2017). Alternatively, a glossy appearance is often associated with very smooth waxy surfaces, which could play a role in water or dust repellence, physical protection or provide a barrier to disease (Vincent and Wegst 2004). Thus, the optical effects produced by smooth structures could be secondary to non-visual functions or of no visual relevance in some taxa. Nonetheless, we are beginning to identify contexts and systems in which gloss does have a visual function. Future research will likely lead to exciting discoveries about how high gloss improves survival of moving prey and in what contexts it is most effective.

## Supplementary Material

arad046_suppl_Supplementary_Material

arad046_suppl_Supplementary_Video

## Data Availability

Analyses reported in this article can be reproduced using the data provided by Henríquez-Piskulich et al. (2023).
