# Supplementary material for: Dazzled by shine: gloss as an antipredator strategy in fast moving prey
Source: Behav Ecol. 2023 Jun 8;34(5):862–71. doi: 10.1093/beheco/arad046 (PMC10516678; doi:10.1093/beheco/arad046)
Supplement: arad046_suppl_Supplementary_Material [file arad046_suppl_supplementary_material.docx]

**Table S.1** Trial order for each group.

| **Group 1 (*n* = 6)** | **Group 2 (*n* = 6)** | **Group 3 (*n* = 7)** | **Group 4 (*n* = 7)** |
| --- | --- | --- | --- |
| 1. Glossy and slow target | 1. Matte and fast target | 1. Glossy and fast target | 1. Matte and slow target |
| 2. Matte and slow target | 2. Glossy and fast target | 2. Matte and fast target | 2. Glossy and slow target |
| 3. Glossy and fast target | 3. Matte and slow target | 3. Glossy and slow target | 3. Matte and fast target |
| 4. Matte and fast target | 4. Glossy and slow target | 4. Matte and slow target. | 4. Glossy and fast target |

**Figure S.1**

**
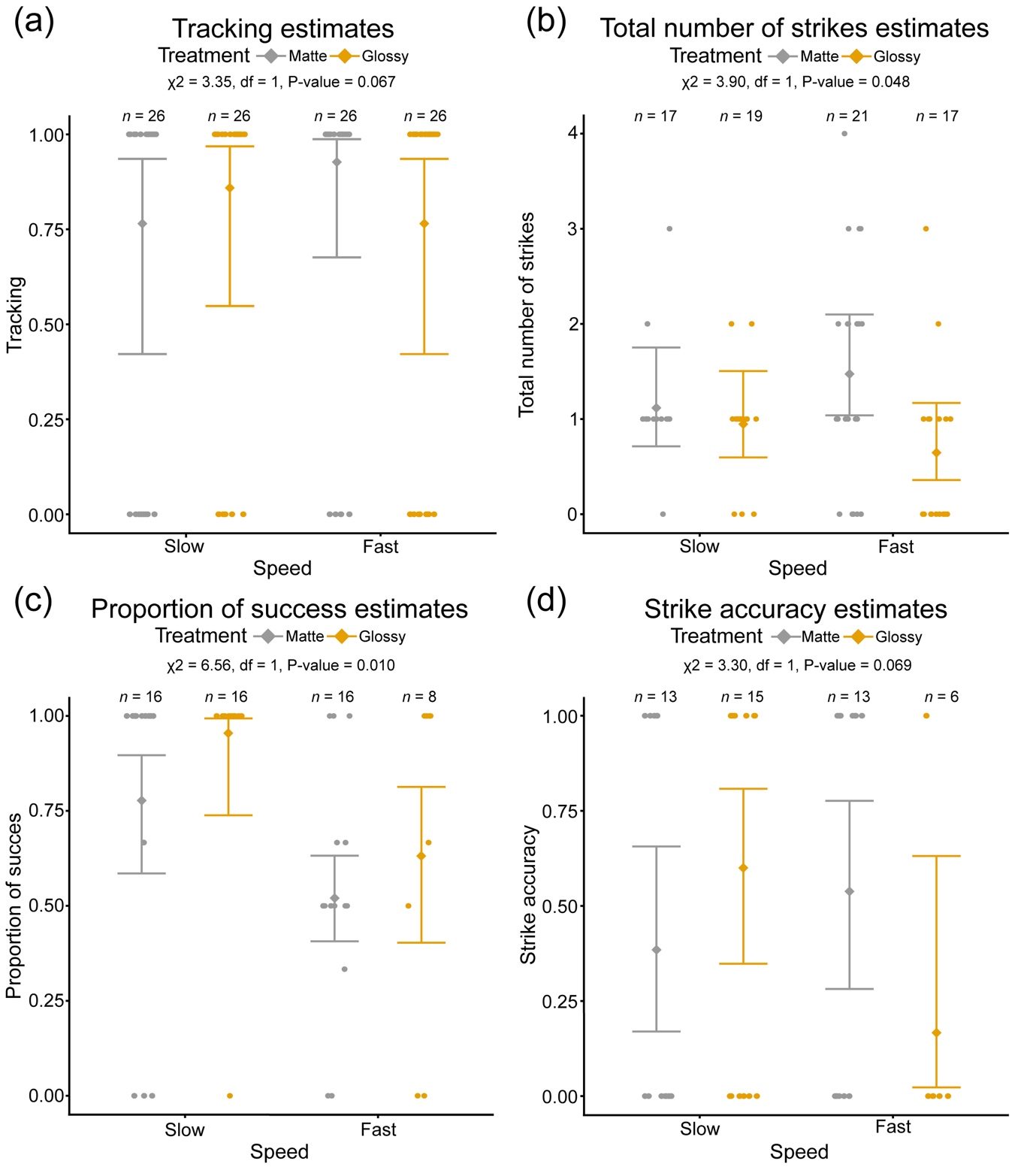
**

Unpooled means and 95% CI estimates from the regression models. Dots represent the observed results of each trial. **(a)** Weak evidence of less tracking for targets that were glossy and fast (interaction term: χ^2^ = 3.35, df = 1, *P*-value = 0.067). **(b)** Moderate evidence that total number of strikes was lowest for glossy targets, regardless of speed (χ^2^ = 3.90, df = 1, *P*-value = 0.048). **(c)** Strong evidence that proportion of successful strikes is lowest at higher speed (χ^2^ = 6.56, df = 1, *P*-value = 0.010). **(d)** Weak evidence of lower strike accuracy for targets that were glossy and fast (interaction term: χ^2^ = 3.30, df = 1, *P*-value = 0.069).
